# Supplementary material for: From sequence to enzyme mechanism using multi-label machine learning
Source: BMC Bioinformatics. 2014 May 19;15:150. doi: 10.1186/1471-2105-15-150 (PMC4229970; doi:10.1186/1471-2105-15-150)
Supplement: Additional file 2 — Java code of ml2db. Additional file ml2db_code.tar.gz contains the Java source code to run the multi-label machine learning experiments and save the results to database. The code’s Javadoc is included. [file 1471-2105-15-150-S2.zip › additional file 2/ml2db/ml2db/doc/main/ml2db/mulan/learn/evaluation/TruePositives.html]

TruePositives


JavaScript is disabled on your browser.


- Overview
- Package
- Class
- Use
- Tree
- Deprecated
- Index
- Help

- Prev Class
- Next Class

- Frames
- No Frames

- All Classes

- Summary:
- Nested |
- Field |
- Constr |
- Method

- Detail:
- Field |
- Constr |
- Method


main.ml2db.mulan.learn.evaluation

## Class TruePositives

- java.lang.Object
- - mulan.evaluation.measure.MeasureBase
  - - mulan.evaluation.measure.BipartitionMeasureBase
    - - mulan.evaluation.measure.LabelBasedBipartitionMeasureBase
      - - mulan.evaluation.measure.LabelBasedFMeasure
        - - mulan.evaluation.measure.MicroFMeasure
          - - main.ml2db.mulan.learn.evaluation.MicroFMeasurePrinter
            - - main.ml2db.mulan.learn.evaluation.TruePositives

- All Implemented Interfaces:
  :   java.io.Serializable, mulan.evaluation.measure.Measure

  ---

    

  ```
  public class TruePositives
  extends MicroFMeasurePrinter
  ```

  Class

  Version:
  :   17 Apr 2012

  Author:
  :   Luna De Ferrari ldf(at)st-andrews.ac.uk or
      luna.deferrari@st-andrews.ac.uk

  See Also:
  :   Serialized Form

- - ### Constructor Summary

    Constructors

    Constructor and Description

    `TruePositives(int numOfLabels)`
  - ### Method Summary

    Methods

    | Modifier and Type | Method and Description |
    | --- | --- |
    | `java.lang.String` | `getName()` |
    | `double` | `getValue()` |

    - ### Methods inherited from class main.ml2db.mulan.learn.evaluation.MicroFMeasurePrinter

      `getFalseNegatives, getFalsePositives, getNumberOfLabels, getTruePositives, setDebug`
    - ### Methods inherited from class mulan.evaluation.measure.LabelBasedFMeasure

      `getIdealValue`
    - ### Methods inherited from class mulan.evaluation.measure.LabelBasedBipartitionMeasureBase

      `reset, updateBipartition`
    - ### Methods inherited from class mulan.evaluation.measure.MeasureBase

      `makeCopy, toString, update`
    - ### Methods inherited from class java.lang.Object

      `equals, getClass, hashCode, notify, notifyAll, wait, wait, wait`

- - ### Constructor Detail


    - #### TruePositives

      ```
      public TruePositives(int numOfLabels)
      ```

      Parameters:
      :   `numOfLabels` -
  - ### Method Detail


    - #### getName

      ```
      public java.lang.String getName()
      ```

      **Specified by:**
      :   `getName` in interface `mulan.evaluation.measure.Measure`

      **Overrides:**
      :   `getName` in class `mulan.evaluation.measure.MicroFMeasure`


    - #### getValue

      ```
      public double getValue()
      ```

      **Specified by:**
      :   `getValue` in interface `mulan.evaluation.measure.Measure`

      **Overrides:**
      :   `getValue` in class `MicroFMeasurePrinter`


- Overview
- Package
- Class
- Use
- Tree
- Deprecated
- Index
- Help

- Prev Class
- Next Class

- Frames
- No Frames

- All Classes

- Summary:
- Nested |
- Field |
- Constr |
- Method

- Detail:
- Field |
- Constr |
- Method
